# ASSOCIATIONS BETWEEN REVERSED CHILDHOOD EXPERIENCE AND LATE-LIFE COGNITIVE IMPAIRMENT: THE CASE IN CHINA

**DOI:** 10.1093/geroni/igad104.1001

**Published:** 2023-12-21

**Authors:** Yalu Zhang

**Affiliations:** Stony Brook University School of Social Welfare, Stony Brook, New York, United States

## Abstract

Exposure to violence, parental incarceration, household substance abuse and some other reversed childhood experience is associated with some negative impacts in one’s late life in some countries, such as difficulties in maintaining interpersonal relationships, being more likely to experience depressive symptoms, and performing certain physical functions. However, the impacts of reversed childhood experiences, such as starvation, unmet needs of healthcare services, and loss of parents, are still unknown. Using nationally representative data, China. Longitudinal Aging Social Survey, and the hierarchical cluster-based partial least squares regression, this study found a notably high prevalence of reversed childhood experience regarding childhood hunger and unmet healthcare needs. In China, 65% of the older adults aged 60 and above had experienced falling asleep with hunger, 83.1% think that their healthcare needs were not met in childhood, and 10.26% lost one or both parents before 10 years old. The regression analysis results show that compared to those who did not have experience falling asleep with hunger, those who had such experience were more likely to have a declined cognitive function by 33.8% (p< 0.001). Additionally, the loss of parents also significantly caused poorer cognitive function by 44.2% (p< 0.001). However, such a causality relationship was not detected among those who experienced unmet healthcare needs. These findings suggest that early prevention and interventions are needed among older adults with adverse childhood experience.

